# The relationship of personality and executive functions in high-level soccer athletes: expertise-and gender-specific differences

**DOI:** 10.3389/fspor.2023.1130759

**Published:** 2023-04-28

**Authors:** Jan Spielmann, Adam Beavan, Jan Mayer

**Affiliations:** ^1^ Department of Sports Sciences, Saarland University, Saarbrücken, Germany; ^2^ TSG ResearchLab, Zuzenhausen, Germany

**Keywords:** team sports, cognition, academy, football, big-five

## Abstract

**Background:**

Psycho-cognitive factors such as personality and executive functions (EFs) are influential parameters when it comes to examining expertise in high-level soccer. Therefore, the profiles of those athletes are relevant both from a practical and scientific point of view. The aim of this study was to investigate the relationship between personality traits and executive functions with age group as an influential factor in high-level male and female soccer players.

**Methods:**

Personality traits and executive functions of 138 high-level male and female soccer athletes from the U17—Pros teams were assessed using the big-five paradigm. A series of linear regressions investigated contributions of personality on EF assessments and team, respectively.

**Results:**

Linear regression models showed both negative and positive relationships between various personality traits, executive function performance and the influence of expertise and gender. Together, a maximum of 23% (*R*^2^ = 6%–23%) of the variance between EFs with personality and various teams, demonstrating that many unaccounted-for variables remain at play.

**Conclusion:**

The results of this study demonstrate the inconsistent relationship between personality traits and executive functions. The study calls for more replication studies to help strengthen the understanding of relationships between psycho-cognitive factors in high-level team sport athletes.

## Introduction

Searching for abilities that help to explain expertise in sport has a longstanding interest in research. Although athletic and physiological factors have largely dominated the research of expertise, psycho-cognitive factors such as personality and cognitive abilities are receiving more attention towards their association with expertise. In the last decade, elite athletes have been demonstrated to yield better general cognitive abilities known as executive functions (EFs; e.g., 1, 2) and further display different expressions of personality traits (3, 4) than their lesser-skilled and non-athletic counterparts. Research has largely examined cognitive abilities and personality traits independently in relation to expertise in sport, but there remains little overlap between these areas. Indeed, these notions have remained relatively distinct concepts in a sporting domain as there has not been a large basis for comparing them. Alternatively, a strong mediating relationship between these psycho-cognitive factors has been reported in domains external to sport. From a neuroscientific approach, both personality and EF constructs are associated with the prefrontal cortex (5). More specifically, the association of working memory and the trait openness rely heavily on the dorsolateral prefrontal cortex (6–8). Despite EFs and personality being conceptually distinct research areas in sport, they are considered to be on a common continuum in other domains with psychology. Therefore, expertise-related literature may benefit from better understanding the psycho-cognitive relationship in a sporting context.

The first construct of interest measuring the core cognitive abilities of athletes, known as their EFs. EFs refer to the family of top-down mental processes that subserve goal directed behaviour (9). EFs are a consciously controlled process that engages in deliberate, goal-directed thought and action (10), and play a role in the decision-making process helping to resolve conflict especially in situations that are new (11). The ability to engage in goal-directed thought and action while negating acting on impulsive decisions can be attributed to the simultaneous development of cognitive control functions such as working memory, inhibition, and flexibility (12, 13). Working memory is responsible for holding information in the mind and findings relationships between the information. Inhibition helps to resist giving in to temptation and preventing acting impulsively and cognitive flexibility allows for the quick and flexible adaptation to changing circumstances or priorities (9). Together, they form the foundation that lower-order cognitive processes such as reasoning, planning or problem-solving rest upon. In a sporting context, EF research falls under the cognitive component skills approach which states that athletes' expertise extends outside a sporting domain and can also be observed in assessments that are decontextualized from their respective sport altogether (14). EFs play a key role in the decision making of athletes (11, 15), helping athletes navigate their environment and ensure that their thoughts and actions remain goal-oriented (10). A recent meta-analysis reported that higher-performing athletes also possess better EFs compared to lower level and non-sporting populations (16) making them an interesting aspect to focus on when relating to high-level athletes.

The second construct of interest for the current study is personality. The dominant theoretical framework for trait personality has been the Five-Factor Model (FFM) (17), also commonly referred to as the Big-Five model (18). The FFM has also been considered the gold standard of personality assessments (19, 20). The FFM framework assesses personality through five broad trait dimensions, consisting of extraversion (reflecting those who are sociable, outgoing, and active), neuroticism (describing individuals who are anxious, hostile, and irritable), openness (distinguishing those who are curious, creative, and imaginative), agreeableness (describing those who are good-natured, unselfish, and forgiving), and conscientiousness (defining those who are organized, punctual, and hardworking).

Outside of the sporting literature, many associations between EFs and FFM have been investigated, as their association explain the mechanisms of individual daily functioning (21). Unsworth et al. (22) reported that openness was moderately related to fluency (i.e., referred to here as cognitive flexibility) demonstrating a link between openness and creative aspects of EF. This is further supported by additional research by Murdock et al. (23), who found significant positive correlations with cognitive flexibility and updating/shifting. Neuroticism, on the contrary, frequently is described to be negatively related to executive and other cognitive functions (4, 24). Results for extraversion are less clear. Some studies found a positive association with shifting/updating (25) and working memory (26), and a negative relationship with behavioural inhibition in children (27) and vigilance (22). Whereas Murdock et al. (23) did not observe a relationship to personality. Although agreeableness has been related to a global EF-score, but there has been no relationship with any specific EF ability when EFs are analysed separately (22, 23). Last, despite conscientiousness being considered as acting with methodical planning and attention, research has yet to find clear relations with EF, which as characterised by being relevant to underpinning these attributes (22, 23). Buchanan (28) found associations of low conscientiousness-levels in children with poor self-report EF but interpreted with caution as the authors did not show any correlations between these self-reported and objective EF measures. A recent study by Johann and Karbach (29) showed a positive relationship between conscientiousness and cognitive flexibility in young adults.

Most research using the FFM rests within the general population whereas the use of this personality assessment in high-performance athletes is still in its early stages (30). The athletic difference between the general population and high-level athletes may yield different relationships between personality and EFs, as high-performing athletes are known to have different cognitive (2) and personality (4, 31) profiles compared to non-athletes. Importantly, as cognitive performance becomes more relevant as the level of competition is greater, this relationship may differ on a function of athlete's expertise levels (4, 14). Vaughan and Edwards (4) were the first to examine the moderating effect of athletic expertise on the link between EF and personality, using computerized assessments of EFs and related them to the FFM. Expertise offset the negative association between neuroticism and EFs, illustrating that although neuroticism was linked to poorer response inhibition, this was not true in the highest expertise group (4). Opposingly, higher neuroticism in more expert athletes lead to greater response inhibition and similar trends were demonstrated in shifting and updating accuracy. This study is however constrained by the sample being comprised of an undisclosed combination of athletes recruited from both interceptive and strategic sports, as per the sport classification system proposed by Voss et al. (14). The study lacks specific information regarding the exact sports included in the sample, as well as the number of athletes sampled from each sport. This omission may hinder the ability to account for potential variations in the relationship between personality traits and EFs across different sports, as prior research has demonstrated that sport type (interceptive, strategic) determines EF differences in elite athletes (32). Consequently, pooling together athletes from diverse sports without considering such complexity may limit the findings and interpretation of the study. Moreover, Vaughan further eluded to the possibility that some of the contrasting findings between EF and FFM compared to previous research in the general population might be due to different age samples, ranging from late childhood [age 9–12 (27)], early adulthood [age 18–27 (4)], and older adults [age 60–85 (33)].

Together, additional research using objective computerized assessments for measuring cognitive abilities is needed (4, 22). Furthermore, narrowing the focus to a single sport eliminates potential confounding variables arising from variations in constraints across different sports, thus allowing for a clearer examination of the relationship between EFs and personality traits. Hence, the aim of this study is to investigate the personality traits of a cohort of elite soccer-only athletes utilizing the Five-Factor Model (FFM), and to establish correlations with each distinct EF ability. A second aim is to understand whether team (across the academy to senior pro teams) is an influential factor in this relationship, similar to how expertise has been reported in sporting populations (4). Therefore, between group differences are examined for each EFs and personality trait. It is hypothesised that high levels of neuroticism will be associated with reduced EF performance, whereas high levels of conscientiousness and openness will be associated with increased EF performance. It is further hypothesised that no relationship between EF and agreeableness or extraversion will exist.

## Methods

### Participants

In total, 138 high-level football players from six teams representing a single high-level German Bundesliga club participated in this study. A total of 44 females were recruited from the senior pro (21.8 ± 2.8) and U20 (17.9 ± 1.3 y) squads, and 94 males were recruited from the senior pro (26.92 ± 4.07 y), U23 (20.8 ± 3.0 y), U19 (17.4 ± 0.6 y), and U17 (16.0 ± 0.2 y) teams. Power analysis (0.80) suggested that a sample size of 98 would be required for linear regression with a medium (0.3) partial eta effect size (G*Power Version 3) (34). One inclusion criterion was that the athletes were German native speakers to prevent the dataset of biases such as misunderstanding the questionnaires or test instructions.

### Personality assessment

The German adaption by Borkenau and Ostendorf (35) of McCrae and Costa (36) NEO-FFI questionnaire was used to determine athletes' personality traits. The questionnaire consists of 60 items rated on a five-point Likert scale (strongly disagree, disagree, neutral, agree, strongly agree). It is a self-report measure that assesses the five personality dimensions: extraversion (E), neuroticism (N), openness (O), agreeableness (A), and conscientiousness (C). The NEO-FFI is a well-established questionnaire with quality criteria reported in various populations [see for further information McCrae and Costa (37)] and in elite soccer players, especially (30). Furthermore, reliability coefficients for the NEO-FFI are shown in Table 1.

**Table 1:** Descriptive statistics, reliability coefficients (alpha), and correlations for all traits.

Trait	All athletes (*n* = 138)						
	Mean	SD	Alpha	N	E	O	A
N	14.47	6.56	.80				
E	31.70	4.51	.62	−.31**			
O	24.60	5.03	.61	.13	.21*		
A	32.20	5.08	.73	−.16	.30**	.05	
C	36.91	5.74	.84	−.37**	.32**	.09	.14

Person correlations for all traits except C (Spearman).

* *p* < 0.05.

** *p* < 0.01.

### Cognitive assessments

All cognitive assessments were run on the Vienna Test System (VTS; Schuhfried GmbH, Austria). The validity and reliability of the VTS has been confirmed by a variety of studies (38–40) and been previously been used in high-level football players (41–43).

#### Determination test

The Determination Test (form S1, Schuhfried GmbH, Austria) is a complex multi-stimuli reaction test involving the combination of five different coloured stimuli and two acoustic signals (2,000 Hz high and 100 Hz low tone) for finger pressing, and two pedal stimuli for the feet. These stimuli corresponded to the pressing of appropriate buttons on the response panel and foot pedals. The Determination Test (DT) aims to measure reactive stress tolerance and the associated reaction speed. The participant must remain composed whilst the quick succession of the single pairing of stimulus and response lasting 4 min. “Correct responses” describes the total number of accurate responses within the 4 min, and “response time” is the median response time (ms) from the appearance of a stimulus to pressing of the correct button. Incorrect reactions are the number of all inappropriate reactions to a stimulus. Omitted Reactions represents the number of stimuli to which no response was made. The validity and reliability of the Vienna Test System has been confirmed by a variety of studies (38–40) and previously been used in high-level soccer athletes (41–43).

#### Response inhibition test

The Response Inhibition Test (form S3; Schuhfried GmbH, Austria), uses a go/no-go paradigm. In each trial, the player is presented either a go-stimulus of a frequent triangle (requires response on green button) or a no-go stimulus of an occasional circle (requires inhibition and no response). In addition, this succession builds up a dominant response tendency because of the similar responses. Each of the 250 of the Inhibition Test (INHIB) stimuli are displayed for 200 ms with an interval of 1 s. The test is displayed in two halves consisting of 101 triangles and 24 circles. The main variable is the number of commission errors, which describes how frequently inhibition of no-go stimuli was unsuccessful. Subsidiary variables represent omission errors which reports the number of omitted reactions to go stimuli; mean reaction time, which is calculated as the mean time for correctly processed go stimuli (44).

#### N-back nonverbal test

The N-back Nonverbal Test (form S2; Schuhfried GmbH, Austria), uses a 2-back paradigm. The player is presented 100 successive stimuli via abstract figures for 1.5 s Abstract stimuli were used, to prevent biases like familiarity of the shown targets in terms of context-specific or intelligence-tendency influences. Players must press a green button, if the actual figure is congruent to the figure, which was presented 2-figures prior. If the figure is incongruent, he does not have to give a response (45). Main variables of the N-back (NBN) are the number of correct reactions to target-stimuli, number of omitted reactions, false positive answers and the mean reaction time for correct responses.

### Procedure

Players conducted one personality questionnaire and three cognitive assessments. All data were measured during a standardised, twice-yearly performance diagnostics event either during preparation time of pre-season (July-August) or mid-season (January-February). The assessments all received a standardized introduction and familiarization protocol and a staff member remained in the test area for consulting and monitoring purposes. Before the participants started, they were informed, that all results would stay anonymous, and they will not get any negative consequence if they do not participate. Testing took approximately 40 min per group accounting for adequate rest between each assessment. Participants did not get any compensation for being part of the study. If required, participants received an explanation of the findings of their individual results via a personal consultation with the club's sport psychologist. Prior to commencement of this study, informed consent for all players was received, and the Institutional Ethics Committee approved this study (approval number: 19-19).

### Statistical analysis

To investigate the contribution of each personality trait on a variety of EF assessments, a series of linear regression models were analyzed. A single model investigated one response variable (i.e., the Determination Test's number of correct responses). There were 11 performance variables of interest, therefore 11 separate linear models were conducted. Each model, neuroticism, extraversion, openness, agreeableness, conscientiousness was added as fixed factors. Furthermore, to account for the known moderation of expertise (4), team was also entered as fixed factor. Each model was run independently for the several parameters provided by the aforementioned tests of cognitive flexibility (DT), inhibition (INHIB) and working memory (NBN) to limit the multi-collinearity associated between EFs. Bonferroni *post-hoc* analyses were conducted where the model reported significant differences between the team. The significance level was set at *p* < 0.05, and an estimate precision was provided using Wald- based 95% confidence intervals. Prior to the analysis, the data were first screened for outliers, missing data, and checked for normality using visual inspection of box plots through a Shapiro-Wilk test of normality in accordance to Tabachnick and Fidell (46).

## Results

Eleven separate linear regression models analyzed the contribution of each personality trait and team on a variety of EF assessment parameters. Collectively, these models indicated that both positive and negative linear relationships exist between various personality traits and performance on EF tests. In other words, each of the five personality traits appeared to have a unique role in either benefiting or hindering EF performance.

Within cognitive flexibility, adding both team and the five personality traits as predictors in the linear regression models provided a significant model fit for response time (*F* = 2.261_10, 123_, *p* = 0.02, *r*^2^ = 16), the number of incorrect responses (*F* = 2.332_10, 125_, *p* = 0.01, *r*^2^ = 16) and the number of omitted responses (*F* = 2.385_10, 123_, *p* = 0.01, *r*^2^ = 16), but not for the number of correct responses (*F* = 1.289_10, 125_, *p* = 0.24, *r*^2^ = 9). These predictors did not improve model fit significantly for the inhibitiońs response time (*F* = 1.227_10, 119_, *p* = 0.28, *r*^2^ = 9) or number of commission errors (*F* = 1.075_10, 125_, *p* = 0.39, *r*^2^ = 8), but was significant for the number of omission errors (*F* = 3.515_10, 120_, *p* = <0.001, *r*^2^ = 23). No model was significant for working memory (*p* > 0.05, *r*^2^ = 6–14). A combination of both personality traits and team explained a maximum of 23% (*R*^2^ = 6–23) of the variance of EF; demonstrating that personality does have an effect on EF performance, but this effect is small. For a further detailed report on the output for each EF variable and the direction to which each personality trait influences each EF parameter, refer to Supplementary Table S1.

### Personality and EFs

Personality did not appear to be strong contributor in the variance associated across most EF assessments. Furthermore, no individual personality trait had a consistent positive or negative contribution across all EF parameters. Furthermore, as detailed below, large confidence intervals exist for all variables demonstrating the widely varied relationships that each personality trait has with performance on the EF-based assessment battery.

Neuroticism was associated with poorer performance on all aspects of cognitive flexibility. For example, for each point increase in neuroticism, the number of incorrect responses increased (0.25 points, CI: −0.29 to 0.80, *p* = 0.36), representing those with higher neuroticism tended to made more incorrect errors. Opposingly, higher neuroticism was linked with better performance in inhibition, where each advancing point in neuroticism decreased reaction time (−0.28 ms, CI: −1.08 to 0.58, *p* = 0.50) and number of omission errors (−0.14 points, CI: −0.48 to 0.20, *p* = 0.42). Performance within working memory varied with higher levels of neuroticism. For instance, each point increase in neuroticism lowered the amount of incorrect responses (−0.06 points, CI: −0.23 to 0.12, *p* = 0.52) and commission error response time (−2.15 ms, CI: −8.15 to 3.85, *p* = 0.48), yet higher neuroticism was also associated with a decrease performance in number of correct responses (−0.02 points, CI: −10 to 0.05, *p* = 0.51), response time (2.84 ms, CI: −1.27 to 6.96, *p* = 0.17) and number of omitted responses (0.02 points, CI: −0.05 to 0.10).

Extraversion was associated with poorer performance on all response variables on cognitive flexibility. For example, for each point increase in extraversion, a decrease in the amount of number of correct responses (−1.52 points, CI: −3.38 to 0.33, *p* = 0.11). Although higher extraversion led to a decrease in inhibition reaction time (0.52 ms, CI: −1.63 to 0.59, *p* = 0.35), and more commission (0.11 points, CI: −0.13 to 0.35, *p* = 0.36) and omission errors (0.18 points, CI: −0.28 to 0.64, *p* = 0.44). Within working memory, higher extraversion was positively associated with lower response times (−4.19 ms, CI: −9.85 to 1.47, *p* = 0.15), but negatively related to number of correct responses (−0.07 points, CI: −0.17 to 0.03, *p* = 0.15) and number of incorrect responses (0.09 points, CI: −0.17 to 0.34, *p* = 0.26).

Openness had a varied effect on performance in cognitive flexibility indicated by the confidence intervals being equally negative and positive. This can be observed in the number of correct responses (0.01 points, CI: −1.56 to 1.58, *p* = 0.99) and number of omitted responses (0.01 points, CI: −0.26 to 0.28, *p* = 0.94). The influence of higher openness was more apparent when assessing inhibition. Each point higher on openness negatively increased reaction time (0.79 ms, CI: −0.16 to 1.73, *p* = 0.10) but reduced omission errors (−0.31 points, CI: −0.71 to 0.08, *p* = 0.44), and the only personality trait to positively reduced the amount of commission errors (−0.24 points, CI: −0.44 to −0.04, *p* = 0.02). Openness was small but positively related to working memory's number of correct responses (0.03 points, CI: −0.05 to 0.12, *p* = 0.41), was both positive and negatively related to slower response time (0.25 ms, CI: −4.58 to 5.08, *p* = 0.92), similar to incorrect and omitted responses.

Agreeableness improved the number of correct responses on cognitive flexibility (0.84 points, CI: −0.85 to 2.53 points, *p* = 0.33) and decreased response time (−0.67 ms, CI: −2.96 to 1.61, *p* = 0.56), there was also a negative relationship to improved number of incorrect responses (0.36 points, CI: −0.33 to 1.04, *p* = 0.31). Agreeableness also decreases reaction time in inhibition (−0.56 points, CI: −1.59 to 0.47, *p* = 0.29) and the amount of omission errors (−0.15 points, CI: −0.57 to 0.28, *p* = 0.5). Respective to working memory, agreeableness improved the number of correct responses (0.09 points, CI: 0 to 0.18, *p* = 0.06), and decreased the number of incorrect responses (−0.17 points, CI: −0.4 to 0.06, *p* = 0.15) and omitted responses (−0.09 points, CI: −0.18 to 0, *p* = 0.06).

Conscientiousness appeared to be positively across all variables on cognitive flexibility, being increases correct responses (0.25 points, CI: −1.20 to 1.70, *p* = 0.73), decreased response time (−0.31 ms, CI: −2.27 to 1.65, *p* = 0.75), and number of incorrect errors (−0.29 points, CI: −0.89 to 0.31, *p* = 0.34). In inhibition, conscientiousness was linked to slower response times (0.37 ms, CI: −0.49 to 1.24, *p* = 0.39), but no clear directional impact on commission or omission errors made. Conscientiousness did lean towards slower response times in working memory (0.48 ms, CI: −4.01 to 4.97, *p* = 0.83), but no direction for commission errors or number of missed responses.

### Team and EFs

Table 2 reports the differences between each personality trait and EF across each team measured. It is observed that the teams of male U19, female U20, and female Pro had similar, yet higher levels of neuroticism compared with the male teams U17, U23 and Pro. Similarly, the two female teams also had the highest levels of agreeableness whereas all the male teams had similar yet lower levels of agreeableness. No noteworthy differences amongst the teams were observed for extraversion and openness. However, differences were observed amongst conscientiousness, where the male U19 reported significantly lower conscientiousness scores compared to the other teams that remained relatively similar.

**Table 2:** Descriptive personality trait and EF statistics across each team (*n* = 138, mean; SD in brackets).

Variable		Females (*n* = 44)		Males (*n* = 94)			
		U20	Pro	U17	U19	U23	Pro
Personality							
Trait	Neuroticism	16.2 (7.1)	16 (5.6)	12.7 (6.5)^a^	17.6 (5.5)^a^^,^^$^	11.9 (5.2)^$^	11.6 (5.1)
	Extraversion	30.7 (5.2)	32.2 (4.6)	32.8 (4.9)	30.0 (3.0)	32.4 (4.7)	31.0 (2.2)
	Openness	24.6 (5.7)	25.6 (5.4)	23.9 (4.1)	24.3 (3.8)	24.5 (5.5)	23.8 (4.1)
	Agreeableness	34.2 (4.8)^#^	35.8 (5.3)^a^^,^^$^	30.8 (4.6)	29.0 (4.9)^a^^,^^#^	31.5 (3.1)^$^	32.5 (3.6)
	Conscientiousness	36.5 (4.2)	37.1 (6.2)	37.8 (5.8)^a^	33.4 (6.0)^a^^,^^$^	38.8 (4.8)^$^	37.3 (4.2)
Executive functions							
Cognitive flexibility	# of Correct	284.4 (39.9)	303.4 (33.0)	300.4 (43.4)	299.7 (36.0)	308.4 (50.3)	272.8 (39.7)
	Response time (ms)	644 (55)^a^^,^^$^	610 (61)	579 (51)^a^	575 (45)^$^	601 (62)	619 (39)
	# of Incorrect	34.8 (16.7)	39.6 (20.1)	37.6 (18.4)	42.5 (15.4)^a^	27.5 (15.9)^a^	28.3 (9.9)
	# of Omissions	16.4 (7.5)	15.2 (6.5)	20.3 (6.4)^$^	19.3 (6.5)^#^	13.3 (8.0)^a^^,^^$^^,^^#^	20.6 (8.1)^a^
Inhibition	Response time (ms)	236 (19)	233 (20)	227 (26)	242 (28)	234 (27)	245 (23)
	# of Commissions	15.7 (5.42)	13.8 (5.13)	15.7 (5.5)	15.0 (6.5)	13.9 (4.5)	13.9 (5.6)
	# of Omissions	9.1 (8.4)	5.4 (6.4)^a^^,^^$^	17.6 (11.9)^a^^,^^#^^,^^&^	17.8 (13.8)^$^^,^^d^	9.4 (9.8)^&^	6.9 (6.5)^#^^,^^d^
Working memory	# of Correct	10.3 (2.4)	11.4 (2.3)	10.8 (2.2)	10.5 (1.9)	10.9 (2.3)	10.3 (2.4)
	Response time (ms)	697 (158)	618 (116)^a^	711 (120)	696 (119)	747 (121)^a^	669 (96)
	# of Omissions	3.7 (2.4)	2.6 (2.3)	3.2 (2.2)	3.5 (1.9)	3.1 (2.3)	3.7 (2.4)
	# of Commissions	7.4 (3.5)	6.1 (4.4)	8.4 (5.9)	9.3 (5.5)	7.6 (4.9)	9.3 (7.4)

^a,$,#,&,d^Symbols represent differences between certain pairs (<0.05).

## Discussion

The aim of the current study was to investigate the personality traits of high-level athletes using the FFM and measure their association to each separate executive function ability. Furthermore, it was also of interest to measure between group differences of this relationship for academy and senior teams. The main finding of the study was that each personality trait did not appear to have a significant positive or negative relationship with the performance variables across the EF battery.

### Relationship between personality and athletic expertise

An early meta-analysis and review-article by Rhodes and Smith (47) reported that respectively physical active people tend to report higher levels in extraversion and conscientiousness and lower levels in neuroticism. Despite a relatively large number of studies measuring with physical active subjects that play sport at a recreational level, there are only a few studies that focus on athletes competing at a high-level. Recently, however, Vaughan and Edwards (4) were the first to investigate the relationship between personality and EF and whether these relationships were moderated by athletic expertise. The researchers recruited individuals with varying levels of expertise, ranging from non-athlete to the super-elite level. Their main finding was athletes scored higher on extraversion, openness, and conscientiousness whereas non-athletes scored higher on neuroticism and agreeableness.

In our current study, using the same personality questionnaire (NEO-FFI) as Vaughan and Edwards (4), we report that the senior male team had the lowest levels of neuroticism, whereas the senior female team reported the highest level of neuroticism compared to the other teams. Extraversion, openness, and conscientiousness remained relatively stable across all teams throughout the youth academy to the adult professional teams. Last, both female teams displayed the highest levels of agreeableness while the senior men's team reporting the highest level across the male teams. The senior men's team displayed the lowest levels of neuroticism is supported by research observing that sport exposes athletes to repeated emotional highs and lows, allowing athletes autonomic nervous system to adapt, leading to lower neuroticism (48). Opposingly, the female Pros displayed one of the highest levels in comparison to the academy teams measures (only male U19 showed higher results). Therefore, it is not fully supported across both senior malés and femalés teams that more experienced athletes have lower neuroticism due to higher accumulated exposure to emotionally taxing sporting experiences in their professional careers. However, as we did not record playing history questionnaires, this possibility is not voided. Furthermore, it was outside the scope of the current study to measure between-gender differences in personality traits. More studies should aim to overcome the gap in literature of between-gender differences in personality traits across the maturation of athletes competing in high-performance sports.

As previously mentioned, the male U19's team reported the highest levels of neuroticism. Moreover, the U19's did report distinct values compared to the other teams in the club. Alongside significantly higher neuroticism levels, the U19's had significantly lower agreeableness and conscientiousness levels. Furthermore, this team also scored the lowest on extroversion, but openness was even with the other teams. This team could be described as less sociable (extroversion), more nervous (neuroticism), less forgiving (agreeableness) and less organized (conscientious) in comparison to the other teams within the club. These findings are contrary to previous studies examining personality traits with playing experience (49) and competitive level in sport (48, 50). This may be a result of the unique constraints of this stage in each athlete's career, where this age group is a highly competitive environment to secure a first team contract within the professional team.

### Relationship between EFs and personality

Vaughan and Edwards (4) reported, that EFs was positively related to openness and conscientiousness, negatively related to neuroticism, bi-directionally related to extraversion, and unrelated to agreeableness. In the current study, we found EFs tended to be negatively related to neuroticism and extraversion, bi-directionally related to openness, and positively related to agreeableness and conscientiousness. However, it must be noted that many of the relationships between each separate personality trait and individual EF performance variable did not reach statistical significance, so although we get an indication of the direction of the relationships, our findings should be interpreted with caution. Furthermore, each personality trait appeared to have both positive and negative relationships with certain variables on the EF assessments, in some traits this was clearer than others. For example, openness was found to be equally positive and negative, with confidence intervals demonstrating that in some cases, it improves, and in others, it hinders EF performance. Vaughan and Edwards (4) reported that EFs was largely positively related to openness, whereas in the current study, higher openness was negatively related to increased response time and higher omission errors in the inhibition test opposing our hypothesis. No clear effect on working memory was observed.

Furthermore, we hypothesized that higher levels of neuroticism would be associated with reduced EF performance, as neuroticism has been shown to be negatively related to EFs in athletes (4). Our findings are partially in line with this notion, where a negative relation between neuroticism and poorer performance on cognitive flexibility and working memory was observed in the form of fewer correct responses, slower response times and higher number of omitted responses. Previous research has demonstrated the susceptibility to experience negative emotions may be expressed as impulsivity, exhibited as an error-prone behavior on performance-based measures of EFs (51). In contrast, neuroticism was also positively related to a decreased response time and fewer omission errors on the inhibition test, and fewer incorrect responses made in the working memory test. The beneficial association observed between neuroticism and inhibition may be a result of the homogenous sample of high-level athletes recruited in the current study. To explain, Vaughan and Edwards (4) reported that although neuroticism was generally linked to worse response inhibition, this was not the case in the more elite athletic groups, where a higher neuroticism with higher expertise led to better response efficiency. Although higher neuroticism is associated with the inability to control desires, perhaps in sport athletes have learned to functionally use impulsivity when quick and firm decisions are required and jumping on opportunities when they seldomly present themselves (52).

Also contrary to our hypothesis, agreeableness appeared to have a positive benefit to performance on the EF assessments. Higher levels of agreeableness improved the number of correct responses and decreased response time on cognitive flexibility and working memory, and decreased response time and omission errors on the inhibition test. This contracts Vaughan and Edwards (4) study where the authors reported no link between agreeableness and EFs regardless of the moderation of athletic expertise. The two female teams did have higher levels of agreeableness than all the male teams, supporting the notion that agreeableness is associated with gender (53). Furthermore, the male professional team had the highest levels of agreeableness. A systematic review indicated that sport participants with high levels of agreeableness report more favorable relationships with their teammates and coaches (54). Opposingly, the younger team in the academy may have lower levels of agreeableness as it may be beneficial for sports achievement where younger athletes may require a higher need competitiveness and aspirations to secure a first team contract (55). This is once more reinforced by the male U19 having the lowest levels of agreeableness.

Extraversion was largely related to negative performance across the EF assessment battery in the form of decreased response times, less total correct answers, and increased errors. For example, on inhibition, extraversion was linked to a decrease in response time but consequently a more erroneous performance. Furthermore, extraversion decreased response time in working memory test but also increased the number of incorrect responses. These results differ with the results reported by Campbell et al. (25), where higher extraversion levels were related to better inhibition and updating ability. Further performance differences were observed with an increase in task difficulty in favor of extroverts. Importantly, Campbell et al. (25) recruited university students and not athletes, and it must be considered that the relationship between EFs changes across the continuum of athletic expertise (4), so direct comparisons are not possible.

Interestingly, conscientiousness had no clear negative or positive relationship with any measured test. Although there was a tendency for the majority of the relationships between conscientiousness and EF variables to be positively beneficial, they too did not reach statistical significance. The weak relationships from the current study do not support the stronger relationships reported by Vaughan and Edwards (4) where higher conscientiousness was associated with better performance across shifting, inhibition and updating or by Johann and Karbach (29) concerning cognitive flexibility. However, conscientiousness levels were similar across all teams in the current study, which is logical given that it is an important predictor of soccer performance over time (56). Conscientiousness is important throughout the career of an athlete as it represents the tendency to control behaviors in service of personal goals (57). This behavioral trait translates into helping athletes stay committed on the development towards expertise and disposition to sustain effort despite adversity. In practice, this could be related to showing up to practice despite exhaustion or soreness (58). However, given the similarity between the goal-oriented role of the conscientiousness trait and that EFs' major role is to ensure one's thoughts and actions remain goal-oriented (9), it is not known why these two variables were not more positively related in the current study.

One similar result between the two studies was that Vaughan and Edwards (4) models explained 13%–27% of the variance between EF with athletic expertise and personality, whereas our models explained 6%–23% of the variance. Together, this represents that although EFs has a relation with the personality of the athlete, there remains many unaccounted-for variables at play. Future studies should aim to explore what other variables can better explain this relationship (48), such as sport-specific (i.e., a contact or non-contact sport, a team or individual sport, etc.) and athlete-specific criteria (i.e., differences in physical body build, sport participation history questionnaires).

The contrasting findings of how personality changed according to athletic expertise may be attributable to the key differences in the methodology between Vaughan and Edwards (4) and the current study. Both studies similarly examined the relationship between athletic expertise, personality and EFs. Vaughan and Edwards (4) recruited athletes from a range of interceptive and strategic sports, but no further details were provide from what actual sports the athletes were sampled from. It remains unknown whether the constraints of each sport has a unique Our current study was a sample of only soccer athletes. This difference is important as both personality (59) and EFs (32) differ between sport type. Furthermore, Vaughan and Edwards (4) evaluated athletic expertise as a range between non-athletic individuals to the super-elite level athlete, whereas our study determined athletic expertise by the progression of the academy teams to the senior adult professional team in a homogenous sample of athletes all competing at the same high-performance soccer club. Last, our results differ from the research because other similar studies have measured EFs using self-reported questionnaires (28, 60, 61), but self-reported and objective measures of EFs have been found to have no relationship (28). Previous methods [apart from Vaughan and Edwards (4)] may have over-simplified the EF construct. Some studies have considered EF a single global construct (33) while others have used separated executive functioning into three core abilities (4). As evidence exists for divergent links between specific EFs and personality, future methodologies should use multiple measures of EF and relate them to individual personality traits rather than a global EF-score (22).

### Limitations and future directions

The findings of the current study should be considered in the context of the study's limitations. First, although the study recruited a large sample of athletes competing at a senior adult professional level, it fell short of the requirements for more complex statistical models that may better capture the complex interrelationships between personality traits and EFs such as structural equation modelling. Future research should look to recruit a larger sample size from a single sport or within the same sport classification to avoid unaccounted for differences in the interactions of personality and EFs that may be unique to each sport. Furthermore, this study did not contain a control group. While the aim of this study was to measure the associations of personality and EFs across different ages in only high-performance populations, not having a control group limits our ability to determine whether the observed relationships between personality and EFs are unique to high-level athletes or generalized to the general population. Last, similar to Vaughan and Edwards (4), the current study was cross-sectional in nature. Future studies should aim employ a longitudinal study with multiple measures to measure the stability of the relationship between measures of EFs and the FFM. Finally, the current study is specific to the athletes competing at the one German soccer academy. Future studies should aim to compare these results with athletes competing at a similar level in various other countries to ensure these findings are more representative to other populations.

## Conclusion

In the current study, we measured whether high level athletes' EFs are predicted by their personality traits. The current study differs from other literature by assessing both male and female soccer athletes using several academy and professional teams. Each personality trait did not appear to have a consistent positive or negative relationship across the cognitive battery. Although one personality trait may demonstrate beneficial improvements in some aspects of executive functioning, each trait tended to equally share negative relationships in other aspects of the EF battery. Therefore, our findings are in line with previous research where personality does appear have a contributable relationship with EF performance, yet this relationship alone underrepresents the true complexity of such relationship. More research is needed on whether the associations between athletes’ EFs and personality reported here can further be supported in other similar athletic groups to help generalize the current study's findings.

## Data Availability

The raw data supporting the conclusions of this article will be made available by the authors, without undue reservation.
