# Supplementary material for: The relationship of personality and executive functions in high-level soccer athletes: expertise-and gender-specific differences
Source: Front Sports Act Living. 2023 Apr 28;5:1130759. doi: 10.3389/fspor.2023.1130759 (PMC10175618; doi:10.3389/fspor.2023.1130759)
Supplement: Supplementary file 1 [file Table1.docx]

**Supplementary Table 1**

A detailed report on the linear model output for each independent variable across both team and personality trait.

| **Variable** | **Estimate** | **CI: 2.5%** | **CI: 97.5%** | **Std. Error** | **t-value** | **p-value** |
| --- | --- | --- | --- | --- | --- | --- |
| *Determination Test: Correct Responses (points)* | | | | |  |  |
| (Intercept) Female Pro | 317.78 | 223.86 | 411.70 | 47.45 | 6.70 | 0.00 |
| Female U20 | -25.35 | -52.01 | 1.32 | 13.47 | -1.88 | 0.06 |
| Male U17 | -0.36 | -24.79 | 24.07 | 12.34 | -0.03 | 0.98 |
| Male U19 | -1.56 | -28.10 | 24.98 | 13.41 | -0.12 | 0.91 |
| Male U23 | 6.05 | -18.57 | 30.66 | 12.44 | 0.49 | 0.63 |
| Male Pro | -32.09 | -62.49 | -1.70 | 15.36 | -2.09 | 0.04 |
| N | -0.21 | -1.55 | 1.13 | 0.68 | -0.31 | 0.76 |
| E | -1.52 | -3.38 | 0.33 | 0.94 | -1.62 | 0.11 |
| O | 0.01 | -1.56 | 1.58 | 0.79 | 0.01 | 0.99 |
| A | 0.84 | -0.85 | 2.53 | 0.86 | 0.98 | 0.33 |
| C | 0.25 | -1.20 | 1.70 | 0.73 | 0.34 | 0.73 |
|  |  |  |  |  |  |  |
| *Determination Test: Response Time (ms)* | | | |  |  |  |
| (Intercept) Female Pro | 639.60 | 513.74 | 765.46 | 63.58 | 10.06 | <0.001 |
| Female U20 | 37.22 | 1.50 | 72.95 | 18.05 | 2.06 | 0.04 |
| Male U17 | -33.47 | -66.22 | -0.72 | 16.55 | -2.02 | 0.05 |
| Male U19 | -40.29 | -76.06 | -4.51 | 18.07 | -2.23 | 0.03 |
| Male U23 | -10.10 | -43.10 | 22.89 | 16.67 | -0.61 | 0.55 |
| Male Pro | 7.99 | -34.00 | 49.98 | 21.22 | 0.38 | 0.71 |
| N | 0.15 | -1.64 | 1.95 | 0.91 | 0.17 | 0.87 |
| E | 0.41 | -2.08 | 2.91 | 1.26 | 0.33 | 0.74 |
| O | -0.41 | -2.51 | 1.69 | 1.06 | -0.38 | 0.70 |
| A | -0.67 | -2.96 | 1.61 | 1.15 | -0.58 | 0.56 |
| C | -0.31 | -2.27 | 1.65 | 0.99 | -0.32 | 0.75 |
|  |  |  |  |  |  |  |
| *Determination Test: Incorrect Responses (points)* | | | |  |  |  |
| (Intercept) Female Pro | -0.14 | -38.54 | 38.26 | 19.40 | -0.01 | 0.99 |
| Female U20 | -1.69 | -12.49 | 9.12 | 5.46 | -0.31 | 0.76 |
| Male U17 | 1.38 | -8.67 | 11.42 | 5.08 | 0.27 | 0.79 |
| Male U19 | 6.61 | -4.24 | 17.47 | 5.49 | 1.21 | 0.23 |
| Male U23 | -8.51 | -18.51 | 1.48 | 5.05 | -1.69 | 0.09 |
| Male Pro | -7.11 | -19.45 | 5.22 | 6.23 | -1.14 | 0.26 |
| N | 0.25 | -0.29 | 0.80 | 0.28 | 0.91 | 0.36 |
| E | 0.84 | 0.08 | 1.59 | 0.38 | 2.20 | 0.03 |
| O | 0.25 | -0.39 | 0.89 | 0.32 | 0.77 | 0.44 |
| A | 0.36 | -0.33 | 1.04 | 0.35 | 1.02 | 0.31 |
| C | -0.29 | -0.89 | 0.31 | 0.30 | -0.97 | 0.34 |
|  |  |  |  |  |  |  |
| **Variable** | **Estimate** | **CI: 2.5%** | **CI: 97.5%** | **Std. Error** | **t-value** | **p-value** |
| *Determination Test: Omission Errors (points)* | | | | |  |  |
| (Intercept) Female Pro | 2.52 | -14.02 | 19.07 | 8.36 | 0.30 | 0.76 |
| Female U20 | 2.17 | -2.47 | 6.82 | 2.35 | 0.93 | 0.36 |
| Male U17 | 5.81 | 1.56 | 10.06 | 2.15 | 2.70 | 0.01 |
| Male U19 | 4.88 | 0.28 | 9.49 | 2.33 | 2.10 | 0.04 |
| Male U23 | -1.06 | -5.35 | 3.24 | 2.17 | -0.49 | 0.63 |
| Male Pro | 6.68 | 1.38 | 11.98 | 2.68 | 2.50 | 0.01 |
| N | 0.15 | -0.09 | 0.38 | 0.12 | 1.23 | 0.22 |
| E | 0.26 | -0.06 | 0.59 | 0.16 | 1.61 | 0.11 |
| O | 0.01 | -0.26 | 0.28 | 0.14 | 0.07 | 0.94 |
| A | 0.05 | -0.25 | 0.35 | 0.15 | 0.33 | 0.74 |
| C | -0.01 | -0.26 | 0.24 | 0.13 | -0.07 | 0.94 |
|  |  |  |  |  |  |  |
| *Response Inhibition Test: Response Time (ms)* | | | | | |  |
| (Intercept) Female Pro | 240.86 | 184.76 | 296.96 | 28.33 | 8.50 | <0.001 |
| Female U20 | 2.49 | -13.62 | 18.61 | 8.14 | 0.31 | 0.76 |
| Male U17 | -8.92 | -23.52 | 5.69 | 7.38 | -1.21 | 0.23 |
| Male U19 | 6.12 | -10.28 | 22.52 | 8.28 | 0.74 | 0.46 |
| Male U23 | -2.73 | -17.45 | 11.99 | 7.44 | -0.37 | 0.71 |
| Male Pro | 9.03 | -8.98 | 27.05 | 9.10 | 0.99 | 0.32 |
| N | -0.28 | -1.08 | 0.53 | 0.41 | -0.68 | 0.50 |
| E | -0.52 | -1.63 | 0.59 | 0.56 | -0.93 | 0.35 |
| O | 0.79 | -0.16 | 1.73 | 0.48 | 1.65 | 0.10 |
| A | -0.56 | -1.59 | 0.47 | 0.52 | -1.07 | 0.29 |
| C | 0.37 | -0.49 | 1.24 | 0.44 | 0.86 | 0.39 |
|  |  |  |  |  |  |  |
| *Response Inhibition Test: Commission Errors (points)* | | | |  |  |  |
| (Intercept) Female Pro | 10.77 | -1.47 | 23.00 | 6.18 | 1.74 | 0.08 |
| Female U20 | 1.88 | -1.59 | 5.35 | 1.76 | 1.07 | 0.29 |
| Male U17 | 2.09 | -1.10 | 5.27 | 1.61 | 1.30 | 0.20 |
| Male U19 | 1.34 | -2.11 | 4.80 | 1.75 | 0.77 | 0.44 |
| Male U23 | 0.60 | -2.61 | 3.81 | 1.62 | 0.37 | 0.71 |
| Male Pro | 0.53 | -3.43 | 4.49 | 2.00 | 0.27 | 0.79 |
| N | 0.14 | -0.03 | 0.32 | 0.09 | 1.64 | 0.10 |
| E | 0.11 | -0.13 | 0.35 | 0.12 | 0.91 | 0.36 |
| O | -0.24 | -0.44 | -0.04 | 0.10 | -2.33 | 0.02 |
| A | 0.09 | -0.13 | 0.31 | 0.11 | 0.79 | 0.43 |
| C | 0.01 | -0.18 | 0.20 | 0.10 | 0.10 | 0.92 |
|  |  |  |  |  |  |  |
| *Response Inhibition Test: Omission Errors (points)* | | | | |  |  |
| (Intercept) Female Pro | 17.70 | -5.78 | 41.18 | 11.86 | 1.49 | 0.14 |
| Female U20 | 3.67 | -3.07 | 10.42 | 3.41 | 1.08 | 0.28 |
| Male U17 | 10.47 | 4.36 | 16.59 | 3.09 | 3.39 | 0.00 |
| Male U19 | 11.27 | 4.53 | 18.02 | 3.41 | 3.31 | 0.00 |
| Male U23 | 2.53 | -3.65 | 8.72 | 3.12 | 0.81 | 0.42 |
| Male Pro | 0.06 | -7.48 | 7.60 | 3.81 | 0.02 | 0.99 |
|  |  |  |  |  |  |  |
| **Variable** | **Estimate** | **CI: 2.5%** | **CI: 97.5%** | **Std. Error** | **t-value** | **p-value** |
| N | -0.14 | -0.48 | 0.20 | 0.17 | -0.81 | 0.42 |
| E | 0.18 | -0.28 | 0.64 | 0.23 | 0.77 | 0.44 |
| O | -0.31 | -0.71 | 0.08 | 0.20 | -1.56 | 0.12 |
| A | -0.15 | -0.57 | 0.28 | 0.22 | -0.67 | 0.50 |
| C | -0.07 | -0.43 | 0.29 | 0.18 | -0.39 | 0.70 |
|  |  |  |  |  |  |  |
| *N-Back Test: Correct Answers (points)* | | | |  |  |  |
| (Intercept) Female Pro | 9.51 | 4.50 | 14.52 | 2.53 | 3.76 | 0.00 |
| Female U20 | -0.82 | -2.25 | 0.60 | 0.72 | -1.15 | 0.25 |
| Male U17 | 0.01 | -1.29 | 1.31 | 0.66 | 0.02 | 0.99 |
| Male U19 | -0.19 | -1.60 | 1.23 | 0.72 | -0.26 | 0.79 |
| Male U23 | -0.08 | -1.39 | 1.24 | 0.66 | -0.11 | 0.91 |
| Male Pro | -0.78 | -2.40 | 0.84 | 0.82 | -0.96 | 0.34 |
| N | -0.02 | -0.10 | 0.05 | 0.04 | -0.66 | 0.51 |
| E | -0.07 | -0.17 | 0.03 | 0.05 | -1.43 | 0.15 |
| O | 0.03 | -0.05 | 0.12 | 0.04 | 0.82 | 0.41 |
| A | 0.09 | 0.00 | 0.18 | 0.05 | 1.94 | 0.06 |
| C | 0.01 | -0.07 | 0.09 | 0.04 | 0.29 | 0.78 |
|  |  |  |  |  |  |  |
| *N-Back Test: Response time (ms)* | | | |  |  |  |
| (Intercept) Female Pro | 641.88 | 355.35 | 928.41 | 144.74 | 4.44 | 0.00 |
| Female U20 | 79.51 | -1.66 | 160.68 | 41.00 | 1.94 | 0.05 |
| Male U17 | 107.06 | 32.50 | 181.62 | 37.67 | 2.84 | 0.01 |
| Male U19 | 70.67 | -11.59 | 152.92 | 41.55 | 1.70 | 0.09 |
| Male U23 | 142.85 | 67.79 | 217.92 | 37.92 | 3.77 | 0.00 |
| Male Pro | 59.52 | -33.06 | 152.10 | 46.77 | 1.27 | 0.21 |
| N | 2.84 | -1.27 | 6.96 | 2.08 | 1.37 | 0.17 |
| E | -4.19 | -9.85 | 1.47 | 2.86 | -1.46 | 0.15 |
| O | 0.25 | -4.58 | 5.08 | 2.44 | 0.10 | 0.92 |
| A | 1.26 | -3.92 | 6.44 | 2.62 | 0.48 | 0.63 |
| C | 0.48 | -4.01 | 4.97 | 2.27 | 0.21 | 0.83 |
|  |  |  |  |  |  |  |
| *N-Back Test: Commission errors (points)* | | | |  |  |  |
| (Intercept) Female Pro | 10.80 | -2.53 | 24.14 | 6.73 | 1.61 | 0.11 |
| Female U20 | 0.98 | -2.46 | 4.41 | 1.74 | 0.56 | 0.57 |
| Male U17 | 1.27 | -1.98 | 4.53 | 1.64 | 0.78 | 0.44 |
| Male U19 | 1.84 | -1.84 | 5.52 | 1.86 | 0.99 | 0.33 |
| Male U23 | 0.53 | -2.77 | 3.84 | 1.67 | 0.32 | 0.75 |
| Male Pro | 2.51 | -1.53 | 6.55 | 2.04 | 1.23 | 0.22 |
| N | -0.06 | -0.23 | 0.12 | 0.09 | -0.64 | 0.52 |
| E | 0.09 | -0.17 | 0.34 | 0.13 | 0.68 | 0.50 |
| O | 0.12 | -0.09 | 0.33 | 0.11 | 1.14 | 0.26 |
| A | -0.17 | -0.40 | 0.06 | 0.12 | -1.46 | 0.15 |
| C | -0.09 | -0.29 | 0.10 | 0.10 | -0.93 | 0.36 |
|  |  |  |  |  |  |  |
| **Variable** | **Estimate** | **CI: 2.5%** | **CI: 97.5%** | **Std. Error** | **t-value** | **p-value** |
| *N-Back Test: Omission Errors (points)* | | | |  |  |  |
| (Intercept) Female Pro | 4.49 | -0.52 | 9.50 | 2.53 | 1.77 | 0.08 |
| Female U20 | 0.82 | -0.60 | 2.25 | 0.72 | 1.15 | 0.25 |
| Male U17 | -0.01 | -1.31 | 1.29 | 0.66 | -0.02 | 0.99 |
| Male U19 | 0.19 | -1.23 | 1.60 | 0.72 | 0.26 | 0.79 |
| Male U23 | 0.08 | -1.24 | 1.39 | 0.66 | 0.11 | 0.91 |
| Male Pro | 0.78 | -0.84 | 2.40 | 0.82 | 0.96 | 0.34 |
| N | 0.02 | -0.05 | 0.10 | 0.04 | 0.66 | 0.51 |
| E | 0.07 | -0.03 | 0.17 | 0.05 | 1.43 | 0.15 |
| O | -0.03 | -0.12 | 0.05 | 0.04 | -0.82 | 0.41 |
| A | -0.09 | -0.18 | 0.00 | 0.05 | -1.94 | 0.06 |
| C | -0.01 | -0.09 | 0.07 | 0.04 | -0.29 | 0.78 |
